# Medicinal Plants as Therapeutic Alternatives to Combat *Mycobacterium tuberculosis*: A Comprehensive Review

**DOI:** 10.3390/antibiotics12030541

**Published:** 2023-03-08

**Authors:** Silvi Gautam, Kamal A. Qureshi, Shabaaz Begum Jameel Pasha, Sugapriya Dhanasekaran, Ashok Aspatwar, Seppo Parkkila, Samyah Alanazi, Akhtar Atiya, Mohd Masih Uzzaman Khan, Divya Venugopal

**Affiliations:** 1 Department of Microbiology, Graphic Era Deemed to be University, Dehradun 248002, India; 2 Department of Pharmaceutics, Unaizah College of Pharmacy, Qassim University, Unaizah 51911, Saudi Arabia; 3 Department of Molecular Analytics, Institute of Bioinformatics, SSE-SIMATS, Chennai 602105, India; 4 Faculty of Medicine and Health Technology, Tampere University, 33520 Tampere, Finland; 5 Fimlab Ltd., Tampere University Hospital, 33520 Tampere, Finland; 6 Department of Clinical Laboratory Sciences, College of Applied Medical Sciences, King Saud University, Riyadh 11362, Saudi Arabia; 7 Department of Pharmacognosy, College of Pharmacy, King Khalid University (KKU), Guraiger, Abha 62529, Saudi Arabia; 8 Department of Pharmaceutical Chemistry and Pharmacognosy, Unaizah College of Pharmacy, Qassim University, Unaizah 51911, Saudi Arabia

**Keywords:** anti-TB compounds, drug-resistant TB, MTB, medicinal plants, natural compounds, phytochemicals, tuberculosis

## Abstract

Tuberculosis (TB) is a serious infectious disease caused by *Mycobacterium tuberculosis* (MTB) and a significant health concern worldwide. The main threat to the elimination of TB is the development of resistance by MTB to the currently used antibiotics and more extended treatment methods, which is a massive burden on the health care system. As a result, there is an urgent need to identify new, effective therapeutic strategies with fewer adverse effects. The traditional medicines found in South Asia and Africa have a reservoir of medicinal plants and plant-based compounds that are considered another reliable option for human beings to treat various diseases. Abundant research is available for the biotherapeutic potential of naturally occurring compounds in various diseases but has been lagging in the area of TB. Plant-based compounds, or phytoproducts, are being investigated as potential anti-mycobacterial agents by reducing bacterial burden or modulating the immune system, thereby minimizing adverse effects. The efficacy of these phytochemicals has been evaluated through drug delivery using nanoformulations. This review aims to emphasize the value of anti-TB compounds derived from plants and provide a summary of current research on phytochemicals with potential anti-mycobacterial activity against MTB. This article aims to inform readers about the numerous potential herbal treatment options available for combatting TB.

## 1. Introduction

History suggests that TB appeared long ago and remained unpredictable until the eighteenth century [1]. It then, at that point, reached pandemic proportions among the increasing population and overwhelming daily situations. In the 20th century, the TB-afflicted population started to reduce rapidly in developed countries with progress in prosperity, food, and dwelling conditions.

TB cases have decreased since the Bacillus Calmette–Guérin (BCG) vaccine was made available in 1921 and antimicrobial drugs such as streptomycin, isoniazid, and rifampicin were made available by prescription in 1943, 1952, and 1963 [2].

MTB infects approximately 8,000,000 people annually, and about 2–3 million die from the disease. It is estimated that 33% of the absolute people are infected with latent TB, with 40% coming from India [3]. With a yearly rate of 2,000,000 new cases, more than 40% of India’s population is infected with latent TB. For about 50 years, counter-TB drugs have been depleted. The emergence of multi-drug-resistant (MDR) and extensively drug-resistant tuberculosis (XDR-TB) strains is a significant challenge in the fight against TB [4]. With the emergence of COVID-19, the number of TB cases has been increasing abruptly, as it has directly impacted TB treatment and diagnosis [5]. With the current situation in mind, the World Health Organization (WHO) has moved the goal of eradicating TB from the world from 2025 to 2035 [6].

The continuous TB treatment methods are only for active tuberculosis and do not address their associated side effects. There has been a general journey for a new source of TB drugs in recent years due to the evolution of multi-drug- resistant TB.

Traditional medicines practiced in South Asia and Africa have indigenous knowledge of medicinal plants and plant-based products that have treated various diseases, including TB. Though these methods may not be able to eradicate the infection completely, they have been known for better management centered on the patient’s quality of life. So, to develop treatment methodologies, upgrade well-being, and address infections with high proportions of morbidity and mortality, alternative methods closest to their natural origins with minimal side effects seem promising [7,8]. Phytodrugs that are plant-based and have a historical pedagogy for treating TB provide an attractive and powerful alternative to conventional treatments with the least side effects [9]. In developing nations, 80% of the population depends on traditional medicine, as indicated [10]. Plants with phytochemicals exhibiting antibacterial activity provide alternatives for treating TB without compromising human health further with side effects [11].

## 2. *Mycobacterium tuberculosis* Overview

### 2.1. Etiology

World Health Organization (WHO) declared TB a global health emergency in 1993. Recently, deaths achieved by TB crossed the number of deaths caused by human immunodeficiency infection (HIV) and gastrointestinal infections together. It is widely believed that over the past two centuries, TB killed one billion people [12].

Tuberculosis, the vitally overwhelming cause of death all over the planet, is a lung infection that spreads when mycobacteria-containing aerosols are formed from spitting, sneezing, coughing, or talking. These aerosol particles are capable of reaching the lower airways. The progression and development of pulmonary TB (which targets the lungs) depends on four stages: phagocytosis of the bacilli, their intracellular proliferation, the latent phase of infection, and lastly, the active form of TB. These stages can result in different clinical outcomes: spontaneous cure, active disease, dormant condition, and reinfection [13].

**Figure 1:**
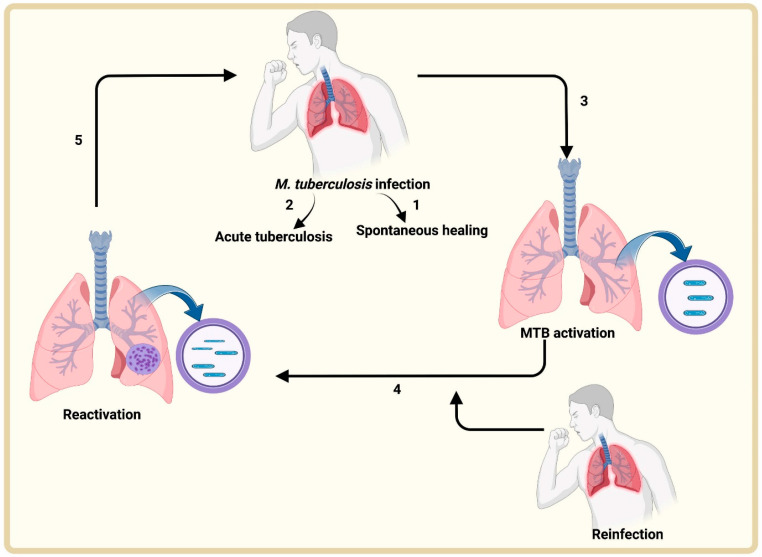
Tuberculosis infection cycle. Air-borne aerosols encapsulating MTB are inhaled by patients. These microorganisms face different outcomes: (1) the pulmonary immune system eliminates MTB; (2) it leads to active TB; (3) the infection progresses to latent TB; (4) the latent TB is reactivated by an endogenous or exogenous means or both; (5) active MTB infection, progression and transmission.

Inhalation of aerosols allows the entry of MTB into the host. Different scenarios are possible (Figure 1):

(1) The pulmonary immune system rapidly eliminates the pathogen;
(2) Infection proceeds to active TB;
(3) The pathogen enters a dormant/latent phase;
(4) The latent MTB can become active following endogenous reactivation, a new exogenous infection, or both;
(5) At this stage, there is MTB spread and transmission.

### 2.2. Epidemiology

According to the Global Tuberculosis Report (2022), progress made in the past to eradicate TB has been slowed down due to the emergence of COVID-19 [6]. COVID-19 virus has had a damaging effect on the treatment and diagnosis of TB. TB cases were high in number in the year 2020 as compared to 2019. America and the East Asian region witnessed a sharp fall in 2020, with a nominal recovery rate in 2021.

There was a clear negative impact of COVID-19 in the European region in 2020, but the reduction was consistent from 2020 to 2021. A marked reduction was observed in 2019 and 2020 in the Eastern Mediterranean region, but it followed up to almost complete recovery in 2021. No recovery was observed in 2021 in the Western Pacific region. Progress in 2021 was higher than in 2019, and the African Region stood out as having only a slightly negative impact in 2020 (−2.3%). The Western Pacific and South-East Asian areas accounted for 84% of the total reduction globally in 2020 and 99% in 2021, respectively, compared to 2019 [6].

Ten countries accounted for most of the global decrease of about 90% in the reported number of people with new TB cases between 2019 and 2020, with India, Indonesia, and the Philippines as the top three countries, accounting for 67% of the total cases [6].

## 3. Current Antibiotic Treatments in Action

### 3.1. Different Antibiotics Used for TB Treatment

The recommended regimen for the treatment of TB involves first-line medications for six months, with an 85% success rate (two months of isoniazid, rifampicin, ethambutol, and pyrazinamide and four months of isoniazid and rifampicin) [14].

First-Line Treatment: There was a significant reduction in TB cases, especially in the developed nations, around the 1950s and 1970s with the discovery of first-line drugs (FLDs) such as isoniazid, rifampicin, ethambutol, pyrazinamide, and streptomycin. Per the guidelines of the WHO, two options are available for drug-susceptible pulmonary TB (DS-TB): a 4-month regimen that includes rifapentine, moxifloxacin, isoniazid, and pyrazinamide or a 6-month course of isoniazid and rifampicin with pyrazinamide and ethambutol in the first 2 months [6].

Second-Line Treatment: A recipe of first and second-line drugs is used for treating MDR and XDR-TB (subject to antibiotic susceptibility testing). The second-line drugs (SLDs) are streptomycin, rifampicin, pyrazinamide, ethambutol, cycloserine, ethionamide, kanamycin, and thioacetazone. Therapy for MDR and XDR-TB is usually more prolonged, less effective, less tolerable, and more expensive than the DS-TB regimen and involves injectable drugs. In a retrospective cohort study, the ratio of patients with MDR-TB who were cured was not more than 69%, even when treated with directly observed treatment [15]. Therefore, new drugs with a novel mechanism or less impact from adverse effects need to be explored to manage drug-resistant TB [16].

### 3.2. Multi-Drug Resistance in Tuberculosis

Drug-resistant (DR) MTB strains identified in communities and medical settings exhibit varying degrees of drug resistance, including rifampicin resistance (RR), MDR, and widespread drug resistance (XDR). TB exhibiting resistance only to rifampicin is identified as RR-TB, while TB showing resistance to isoniazid and rifampicin is known as MDR-TB [17]. The treatment of RR-TB and MDR-TB is longer than 18 months and usually consists of first-line drugs and selected second-line drugs. In XDR-TB, apart from resistance to isoniazid and rifampicin, there is resistance to at least one drug from the two classes of second-line drugs—fluoroquinolones and injectables. This implied that there is resistance to moxifloxacin or levofloxacin among the fluoroquinolones and amikacin, kanamycin, or capreomycin among the injectable agents [18]. Multi- and extended-drug resistance have been caused by a variety of factors, including non-compliance with therapy, failure to directly observe treatment, shortage of drugs, poor quality of drugs, access to anti-TB drugs without a prescription, poor medical management, and non-adherence to national control programs [19].

In 2018, approximately a million instances of drug-resistant tuberculosis (DR-TB) were reported, with 78% of those cases being MDR-TB [14]. MDR, extensively drug-resistant (XDR), and drug-resistant are the three types of drug-resistant MTB strains identified by the WHO. Isoniazid and rifampicin, the two first-line drugs, are ineffective against MDR-TB, which makes up around 3.4% of new TB cases worldwide. MDR-TB requires a lengthy, expensive course of therapy lasting 9 to 20 months, with a 56% slower success rate than unprotected TB [14]. Rifampicin and isoniazid are also ineffective against XDR-TB, as are fluoroquinolones and one of the three second-line injectable drugs (kanamycin, capreomycin, or amikacin), with a lower rate of success of 39% [14]. Finally, no medicine can penetrate XXDR strains. XXDR strains are resistant to all first and second-line drugs. There has been research using atomic force microscopy to study XXDR-MTB strains in the exponential phase that observed changes in cell morphology, including increased roughness and striations and tubular extensions. These alterations are likely due to drug treatment, and about 5–7% of the XXDR-MTB bacteria displayed an unusually thick cell envelope, regardless of the strain genotype [20].

The chemical-based antibiotics discussed above are well established, have been in clinical use for treating TB for a long time, and have been recommended by the WHO. Most of these antibiotics inhibit the growth of bacteria mainly by interfering with ribosomal proteins, transcription, and translation of proteins. Pathogenic bacteria, specifically MTB, have developed various levels of resistance to these antibiotics. Therefore, medicinal chemists and researchers in anti-TB drug development are searching for novel compounds that target pathways other than the mechanisms targeted by clinically used antibiotics. In the recent past, the focus has been on developing small molecular inhibitors that target novel pathways of MTB that are crucial for the survival of the bacteria, and MTB has not developed resistance to these so far.

When a pathogen enters the host, it encounters stressful conditions, such as acidic pH, free radicals, hypoxia, and nutrient starvation. It has been shown that MTB contains three β-carbonic anhydrases (β-CAs) required for the survival of the bacterium in the harsh environment of the host. The role of CAs is well established in pH homeostasis and the bicarbonate transport required for the cell’s metabolic activities [21,22]. In mycobacteria, the β-CAs are known to play a crucial role in biofilm formation and the production of virulence factors and hence survival and tolerance of antibiotics [23,24]. There has been an effort to develop small molecular compounds targeting the β-CAs of mycobacterium as anti-TB compounds [22,25].

Interestingly, humans lack β-CAs and contain only α-CAs; therefore, the anti-TB compounds that target the β-CAs of MTB cause minimal side effects and potentially treat drug-resistant TB. It has been demonstrated that dithiocarbamates specifically inhibit β-CAs and thus inhibit mycobacterium growth in vitro and in vivo in the zebrafish model [26]. In addition to chemically synthesized CA inhibitors, some plant-derived compounds known as coumarins (Table 1) have been shown to exhibit antibacterial and anti-tubercular activity [27,28]. Coumarins inhibit the synthesis of proteins and suppress the activity of carbonic anhydrases [27,28]. It has recently been shown that coumarins are involved in microbial quorum sensing and biofilm formation [29].

Table 1 shows the anti-TB antibiotics and carbonic anhydrases and their mechanism of action [16].

## 4. Drug Delivery Incorporating Nanoparticles for the Treatment of TB

**Figure 2:**
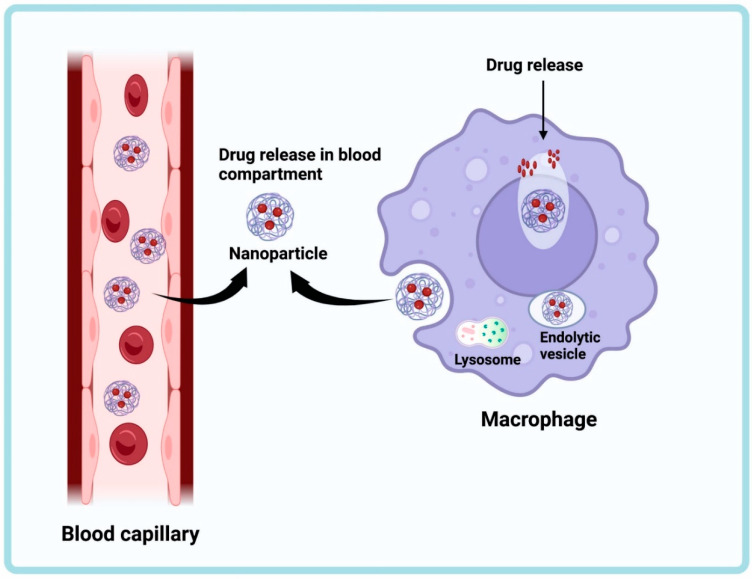
Mechanism of drug delivery by nanoparticles. The encapsulated drug release occurs in the infected macrophages. The drug is released in the blood compartment of the capillary. The nanoparticles from the blood capillary are engulfed by the infected macrophage, and the drug is released inside.

Nanotechnology-based medicine delivery systems have significantly improved healthcare and made it feasible to fight illnesses at the molecular level [30]. Nanoparticles are elegant vehicles for drug-targeted delivery because of their great stability and carrier capacity (Figure 2) [31,32]. In India, which has the greatest estimated number of TB cases, research is being performed to evaluate what role nanotechnology can play in fixing these issues [33].

Biocompatible and biodegradable components of nanoparticles include materials such as polymers, which can be either natural (such as gelatin and albumin) or synthetic (such as polylactides and polyalkylcyanoacrylates) or solid lipids (SLNR and NLCR) [34].

The utilization of nanoparticles for delivering drugs presents various benefits such as extended stability resulting in longer shelf life, significant capacity to carry drug molecules, the possibility of encapsulating both hydrophilic and hydrophobic substances, and versatility in administration methods, including oral intake and inhalation [30].

Antimicrobial efficacy has been further enhanced by synthesizing silver nanoparticles (AgNPs) mediated by an endophytic fungus such as *Aspergillus fumigatus*. Bactericidal activity was significant against *Staphylococcus aureus*, *Shigella dysenteriae*, and *Salmonella typhi* by damaging the cell membrane and releasing the cellular contents [35].

## 5. Drug Delivery Explored in TB Treatment

Liposome-Based Drug Delivery Systems: Aqueous and phospholipid bilayers are bridged to form tiny, closed vesicles known as liposomes. Due to their unique capacity to encapsulate hydrophilic and hydrophobic medications, they have received extensive study as a viable drug delivery platform for bioactive chemicals. In both in vitro and in vivo testing, liposomes considerably lowered the drug’s toxicity and demonstrated improved anti-TB activity in both acute and chronic scenarios [34]. Mice with persistent infection administered liposome-encapsulated PZA (pyrazinamide) and RFB (rifabutin) exhibited complete bacilli clearance from the lungs, liver, and spleen [36,37].

Nanoemulsion-based Drug Delivery Systems: Nanoemulsions have received a lot of attention recently for the study and development of novel drug delivery systems due to their thermodynamic stability, high diffusion and absorption rates, ease of production, and high solubility [31,32,38].

Solid Lipid Nanoparticles (SLNs)-Based Drug Delivery Systems: SLNs are water-based nanocrystalline nanosuspensions that can be ingested. They have several benefits over polymeric nanoparticles and liposomes, including increased stability and more effective encapsulation [39]. SLNs have improved encapsulation efficiency, longer or higher stability, and require fewer organic solvents than polymeric nanoparticles and liposomes [34].

Polymeric-nanoparticles-based Drug Delivery Systems: Depending on the preparation technique, polymeric nanoparticles are referred to as nanoparticles, nanospheres, or nanocapsules because the drug is connected to, entrapped in, or enclosed in the polymeric core [40]. Five oral dosages of nanoparticles every ten days were sufficient to eliminate TB bacillus from the organs of mice, whereas free medicines required 46 treatments to obtain the same outcomes.

Alginate-Based Anti-TB Drug Delivery System: Alginate is a naturally occurring biopolymer that has seen increased use in various industries. It performs multiple functions, including tablet disintegration and binding, gel, cream, lotion solidification and suspension, and emulsion stabilization [34]. The formulation provides a prolonged release in the plasma for 7–11 days and in the organs for 15 days after a single oral dose [41].

Kaur et al. demonstrated the development and characterization of guar gum nanoparticles for oral immunization against TB [42]. Using ethanol as an antisolvent, guar gum nanoparticles were created during the solvent’s precipitation. The solvent’s composition was changed, with the amounts of ethanol and water added in drops to the guar gum solution containing the antigen. Several formulation parameters, including polymeric concentration and solvent composition, were improved based on particle size and distribution. It was concluded that other factors were held constant, such as the type of solvent used, the rate of stirring, the temperature, and the rate of addition. It was found that the polymer concentration can significantly affect the size of the particles in a formulation. The guar gum nanoparticles triggered a potent immune response on both the systemic and mucosal levels. As a result, guar gum nanoparticles were found to be a valuable tool for delivering targeted vaccinations without needles. They can deliver oral vaccines safely and effectively [4]. Another study [43] concluded that zinc oxide nanoparticles (ZnO NPs) could be synthesized using the aqueous extract of *L. acidissima* L. leaf. These nanoparticles restrict MTB development, and the microplate alamar blue method was used to validate this. Biogenic ZnO NPs have the potential to become an innovative drug component to treat TB. In another investigation by Murali et al., Zn NPs synthesized using *Canthium dicoccum* (L.) plants for antibacterial, anti-tubercular, and anti-oxidant action were validated [44]. According to this report, the synthesis of ZnO NPs using leaf extract from *C. dicoccum* (L.) was successful, resulting in pure nanoparticles within the desired size range. This was confirmed through the use of several techniques, including UV-Visible spectrophotometry, dynamic light scattering (DLS), scanning electron microscopy (SEM), transmission electron microscopy (TEM), and X-ray diffraction (X-RD). It was found that the Zn NPs exhibited strong antibacterial and anti-mycobacterial activity; the data suggest the vulnerability of tuberculosis strains to ZnO NPs [44]. Some plants have already been used for treating MTB infection using nanoparticles (Table 2) [45].

## 6. Phytoproducts: An Emerging Alternative

The antibiotics that have been widely used for the treatment of TB leave some major side effects on the patients treated, such as:

Streptomycin sometimes leads to renal damage and vestibular and auditory nerve damage; isoniazid may cause hepatitis; rifampicin causes thrombocytopenia, pain, vomiting, nausea, and hepatitis; pyrazinamide leads to arthralgia and hepatitis; ethambutol may cause neuritis and color blindness; cycloserine results in convulsions, dizziness, depression, and psychotic reactions; ethionamide causes diarrhea, abdominal pain, and hepatotoxicity; and kanamycin may cause vertigo, damage to the auditory nerve, nephrotoxicity, skin rash from thioacetazone, and exfoliative dermatitis [63]. Researchers are working on alternate medications—phytoproducts—to avoid such side effects.

Traditionally, medicinal plants were used for treating TB, such as smoke from the burnt leaves of *Artemisia afra*, the whole plant of *Myrothamnus flabellifolius*, leaves of *Carica papaya*, *Zanthoxylum capense* (roots), and seeds of *Combretum hereroense*, which was was inhaled 3–4 times every day. Leaves of *Artemisia afra* and *Lippia javanica* were infused into a hot water sink and patients inhaled the steam by wrapping a blanket over their heads. Leaves of crushed *Citrus lemon*, *Artemisia afra*, and *Mentha* sp. were burned in a paper wrapper 2 or 3 times daily. Such ayurvedic treatments are administered for around two weeks up to a month, subject to the patient’s reaction to the formulation and tolerance to the medicine and administration [64,65].

Medicinal plants contain phytochemicals that are primary and secondary products of metabolism [66]. There is an abundance of studies available in the literature regarding the biotherapeutic purposes of these metabolites that fall into different classes of compounds, such as flavonoids, carotenoids, indoles, isothiocyanates, monoterpenes, and phenolic acids [67]. The regimen of the cocktail of antibiotics administered for the treatment of TB is globally known as the “Directly Observed Treatment Short-course (DOTS)”. Its severe toxicity and side effects are established [68]. Infection by MTB leads to suppression of the Th1 response, leading to reduced levels of proinflammatory cytokines and high levels of anti-inflammatory cytokines [69]. Due to the reduction in protective CD^4+^ T cell numbers and the side effects of DOTS therapy, patients are prone to reinfection and reactivation of infection [70]. The overall efficacy of the anti-tubercular treatment is increased by using anti-inflammatory drugs along with the standard regimen [71]. However, the long-term use of these drugs results in severe side effects, making such treatments inadvisable. Therefore, researchers are exploring molecules that can immunomodulate by selectively upregulating the Th1 response and simultaneously downregulating Th2 immune response [72].

Plant-based compounds offer potential candidates for immunomodulators. The alcoholic extract of *Coleus scutellarioides* (Miana leaves) immunomodulates by upregulating the proinflammatory T-lymphocyte response and increasing the levels of IFN-γ and TNF-α [73]. Bergenin, a secondary metabolite found in different parts of several plants [74], induces Th1 and Th17 response and inhibits bacterial replication in murine models [75]. Further, co-treatment of bergenin and isoniazid reduces the immune damage caused by isoniazid while promoting the generation of long-lasting, central memory T-cell responses and reducing MTB clearance duration [76]. Similarly, silymarin isolated from *Sylibym marianum* seeds induces an elevated Th1 response in drug-sensitive and drug-resistant strains [77]. Immunomodulators of plant-based origin include allicin from garlic; piperine, an extract of *Chanca piedra* (*Phyllanthus niruri*); curcumin from turmeric; gingerol from ginger; and extracts of Rubiaceae species, which also act as anti-oxidants and anti-inflammatory agents [78]. They may be used synergistically as adjuncts to DOTS as alternatives to steroids for managing inflammation.

**Figure 3:**
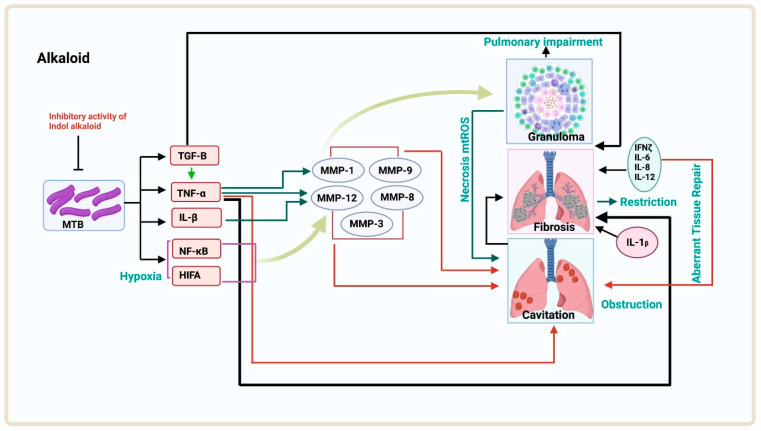
The isolated compound from the plant shows inhibitory activity on MTB. The MTB cells in hypoxia conditions produce HIFA, NF-κB, IL-1B, and TNF-α, and these proteins lead to the production of MMP-12, MMP-3, MMP-1/MMP-8, and MMP-9 inhibitors. The production of these matrix metalloproteinases leads to cavitation in the lungs, or MMP-12 and MMP-1 together produce granuloma, which leads to pulmonary impairment or necrosis, leading to cavitation followed by obstruction. Cavitation causes fibrosis in between the mechanism of aberrant tissue repair.

**Figure 4:**
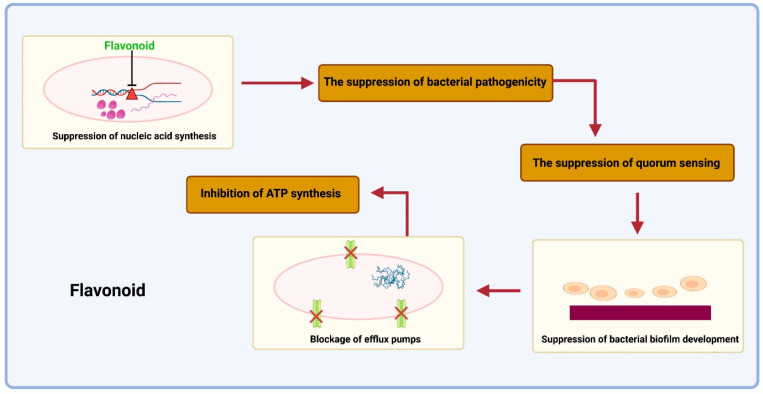
The detrimental suppression of bacterial pathogenicities by flavonoids. Toxins, quorum sensing, dihydrofolate reductase (DHFR), helicases, and gyrase/topoisomerase inhibitors suppress nucleic acid synthesis. The capacity to produce biofilms then inhibits the synthesis of the cell envelope. Subsequently, it involves blocking the enzyme fatty acid synthase (FAS—5) and peptidoglycan production (suppression of Ala-Ala dipeptide synthesis, inhibition of peptidoglycan cross-linking). Additionally, flavonoids can block efflux pumps, which may result in the reversal of antimicrobial resistance. Then, the bacterial respiratory chain’s ATP synthase and NADH-cytochrome c reductase activities are inhibited.

**Figure 5:**
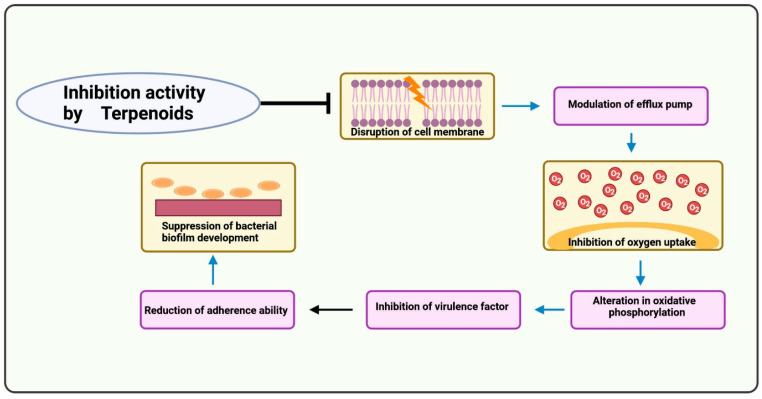
Mechanism of action of terpenoids. Terpenoids disrupt the cell membrane. The efflux pump is modulated, which might reverse antimicrobial resistance. The oxygen uptake process is blocked, resulting in the inhibition of oxygen. The oxidative phosphorylation process is altered, inhibiting other virulence factors. The fatty acid synthase enzyme and peptidoglycan production are blocked, suppressing biofilm production.

Many phytocompounds have already been isolated in the past and found to have anti-TB activity. These products include alkaloids, flavonoids, and terpenoids [79]. The basic mechanism of these photoproducts is shown below (Figure 3, Figure 4 and Figure 5):

Table 3 shows the plants with known anti-tubercular activity. Their anti-tubercular principles have yet to be identified, whereas Table 4 shows the plants whose bioactive compounds have already been isolated for the treatment of TB.

## 7. Conclusions and Future Perspectives

Medicinal plants and plant-based products provide an attractive alternative for not only eliminating bacteria but also as potential adjuvants for alleviating the side effects of standard anti-mycobacterial drugs. With the rise of drug-resistant strains of TB, the need to explore alternative remedies has become more pressing than ever before. As this article highlights, India’s traditional medical philosophies offer a treasure trove of knowledge and practices that can be harnessed to develop new and effective treatments for TB. The search for phytodrugs is an important step towards better management of TB. Despite the growing focus on the biotherapeutics of phytochemicals in various diseases, this has lagged in the area of TB treatment. More studies are needed regarding mechanism of action, potential for bacterial clearance, and immunomodulatory effects. By developing a multidisciplinary approach to include promising candidates of phytodrugs as adjuvants in medical treatment, we can potentially alleviate the problem of drug resistance, improve the efficacy of DOTS therapy, and improve the overall patient outcome. This strategy is particularly significant considering the limited repertory of antibiotics and the developing forms of resistance in TB.

Using plants and plant-based products in TB treatment offers an exciting opportunity to design new treatments and cures for better health management. As it is known that DOTS, while clearing bacteria, suppresses the host immune system, phytochemicals as an adjunct drug could boost immune cells and offer a promising alternative to traditional antibiotics, thus providing an avenue for developing sustainable and environmentally friendly treatments.

However, it is essential to note that alternative medicines must be used with caution. While traditional remedies may offer potential benefits, they must be rigorously tested to ensure their safety and efficacy. Scientific studies must be conducted to investigate the use of phytodrugs in TB treatment to ensure that patients receive the best possible care. New molecules such as sequella (SQ109), benzothiazinones (BTZ043), and piperazine-benzothiazole (PBTZ169) with a similar pharmacophore to the phytochemical piperine are undergoing clinical trials [115].

In conclusion, using local medications, beneficial herbs, and their constituent parts in TB treatment is a promising area of research. India’s traditional medical philosophies offer a wealth of knowledge and practices that can be harnessed to develop new and effective treatments for TB. Exploring the potential of phytodrugs and other plant-based products that can reduce the side effects of standard drugs in synergistic combinations may improve the overall outcome for patients and develop sustainable, environmentally friendly treatments. With further research and development, we can look forward to a future where TB is no longer a major threat to public health.

## Figures and Tables

**Table 1:** Anti-TB drugs and their mechanism of action.

Drugs	Mode of Action	References
Streptomycin	Reduction in the production of ribosomal proteins	[14]
Isoniazid	Cellular, lipid, carbohydrate, and NAD metabolism inhibition	[14]
Pyrazinamide	Membrane transport disruption and energy exhaustion	[14]
Rifampicin	Inhibiting the synthesis of RNA	[14]
Cycloserine	Reduction in the production of mycolic acid	[14]
Kanamycin	Decrease in protein synthesis	[14]
Ethambutol	Inhibiting the synthesis of RNA	[14]
Quinolones	Inhibition of transcription replication and DNA replication	[14]
CA inhibitors	Inhibit the activity of carbonic anhydrases needed for pH regulation	[21,23,24,25,26]
Coumarins	Inhibit protein synthesis and activity of carbonic anhydrases	[27,28]

**Table 2:** Plants and their parts used in TB treatment.

Scientific Names	Plant Part Used	NP Used	Size and Shape	Ref.
*Nerium oleander*	Leaf extract	Gold NP	2–10 nm, spherical	[46]
*Butea monosperma*	Bark	Gold and silver NP	10–100 nm, spherical or triangular	[47]
*Pea nut*	Skin and seed	Gold NP	110–130 variable	[48]
*Hibiscus cannabinus*	Stem extract	Gold and silver NP	10–13 nm, spherical	[49]
*Sesbania grandiflora*	Leaf extract	Silver NP	7–34 nm, spherical	[50]
*Salix alba*	Leaf extract	Gold NP	50–80 nm, non-spherical	[51]
*Eucommia ulmoides*	Bark	Gold NP	Not known	[52]
*Galaxaura elongata*	Powder or extract	Gold NP	3.85–77.13 nm, spherical, triangular, and hexagonal	[53]
*Ocimum sanctum*	Leaf extract	Gold and silver NP	30 nm, hexagonal	[54]
*Torreya nucifera*	Leaves and bark	Gold NP	10–125 nm, spherical	[55]
*Olea europaea*	Leaf extracts	Gold NP	50–100 nm, triangular and hexagonal	[56]
*Rosa indica*	Rose petals	Gold NP	3–15 nm, spherical	[57]
*Pistacia integerrima*	Galls extract	Gold NP	20–200 nm	[58]
*Terminalia arjuna*	Fruit	Gold NP	60 nm, spherical	[59]
*Euphorbia hirta*	Leaf extract	Gold NP	6–71 nm, spherical	[60]
*Morinda citrifolia*	Root extract	Gold NP	12.17–38.20, spherical	[61]
*Zizyphus mauritiana*	Extract	Gold NP	20–40 nm, spherical	[62]

**Table 3:** Plants with known anti-tubercular activity. Their anti-tubercular principles are yet to be identified.

Plant	Part Utilized	References
*Aframomum melegueta*	Roots	[80]
*Artemisia sativa* L.	Leaves	[81]
*Cannabis sativa* L.	Leaves	[82]
*Carica papaya*	Leaves	[83]
*Chironia baccipfera*	Roots	[81]
*Combretum hereroense* Schinz	Bark	[84]
*Citrus lemon*	Leaves	[85]
*Eucalyptus camaldulensis*	Leaves and root	[86]
*Eucomis pallidiflora* Baker. ssp. pole-evansii	Bulb	[87]
*Hypoxis hemerocallidea*	Tuber	[88]
*Lippia javanica*	Leaves	[84]
*Merwilla plumbea*	Bulb	[82]
*Myrothamnus flabellifolius*	Whole plant	[84]
*Salix mucronata*	Seeds	[89]
*Zanthoxylum capense*	Roots	[90]

**Table 4:** Isolated bio-active compounds with anti-tubercular activity.

Plant	Section	Bioactive Compounds	Chemical Structure	Ref.
*Allium sativum* L.	Bulb	Peptides, ajoenes, vinyldithiins, alliin, allicin, 𝛾-glutamylcysteine, sulfides	𝛾-glutamylcysteine, peptides, alliin, ajoenes	[91]
*Annickia chlorantha* (Oliv.) Setten and Maas	Stem bark	Berberine and protoberberine alkaloids: columbamine, palmatine, jatrorrhizine	Not known	[92]
*Aframomum melegueta*	Roots	Tannins	Not known	[80]
*Aristolochia taliscana*	Roots	Licarin A	Licarin A	[93]
*Aloe barbadensis*	Sap leaves	Anthraquinone glycosides: emodin, barbaloin; enzymes, sugars, lignin, saponins, minerals, salicylic acids, galacturonic acid, vitamins	Emodin and barbaloin	[94]
*Bacopa monnieri*	Stem and leaves	Brahmine, herpestine, nicotine, D-mannitol, monnierin, bacosides a, hersaponin, saponins B, saponins C, bacosides B, saponins A, betulinic acid, stigmasterol	Nicotine	[95]
*Phellodendron amurense* Rupr.	Bark	Isoquinoline alkaloid	Isoquinoline	[96]
*Curcuma longa* L.	Whole plant	Mono-O-methylcurcuminisoxazole (chemically modified curcuminoid analog)	Not known	[97]
*Celastrus vulcanicola*	Leaves	1α-acetoxy-6β,9β dibenzoyloxydihydro-β-agarofuran	Not known	[98]
*Diospyros anisandra*	Stem bark	Maritinone and 3,3′- biplumbagin (dimers of plumbagin)	Maritinone and biplumbagin	[99]
*Foeniculum vulgarevar*	Stem and leaves	2,4-undecadienal	2,4-undecadienal	[100]
*Greenwayodendron suaveolens*	Stem	Polysin, indolosesquiterpenes, greenwayodendrin-3-one, 3-O-acetyl greenwayodendrin, N-acetylpolyveoline, polyveoline, aporphines	Not known	[101]
*Justicia vasica*	Leaves, roots stem, bark	Adhatodine, vasicinone, anisotine, vasicine, vasicoline, sicinolone, vasicolinone	Adhatodine, vasicine, vasicinone, and vasicolinone	[95]
*Leucophyllum frutescens*	Root	Leubethanol	Leubethanol	[102]
*Lippia javanica*	Leaves	Flavonoids	Quercetin	[84]
*Lannea nigritana* (Sc. Elliot) Keay	Root	Tannins and phenolic compounds (Lanneanol)		[103]
*Morinda citrifolia*	Whole plant	Anthraquinones: morindone, glycosides: flavanol and iridoid, triterpenoids β-sitosterol, ursolic acid, asperuloside, carminic acid, damnacanthol	Morindone and damnacanthol	[104]
*Mentha piperita*	Leaves	Essential oils, menthol, menthone and menthofuran	Menthol, menthone, and menthofuran	[105]
*Ocimum tenuiflorum*	Leaves	Essential oil consists mostly of: eugenol (~70%) germacrene, isothymusine, β-bisabolene (13–20%), β-elemene, caryophyllene, methyl chavicol (2–12%), 1,8-cineole (9–33%)	Eugenol, germacrene, and caryophyllene	[106]
*Pupalia lappacea Juss.*	Leaves	Alkaloids, glycosides, saponins, tannins, starch, coumarins, terpenoids, and steroids such as 1-docosanol, amino acids, glycosides, flavonoids, stearic acid, stigmasterol, sitosterol, sitosterol-3-O-D-glucopyranoside, stigmasterol-3-O-D-glucopyranoside, N-benzoyl-L-phenylalaninol acetate, and 20-hydroxyl ecdysone	Setosterol-3-O-D-glucopyranoside, N-benzoyl-L-phenylalaninol acetate, sitosterol-3-O-D-glucopyranoside	[107]
*Plectranthus fruticosus* L.	Whole plant	Abietane and its derivatives 6,12-dibenzoyl, 12-nitrobenzoyl esters, 12-chlorobenzoyl, 12-methoxy benzoyl	Abietane and 12-methoxy benzoyl	[108]
*Plumeria Bicolor*	Bark	Plumericin and isoplumericin	Plumericin and Isoplumericin	[4]
*Plumbago indica* L.	Root	Plumbagin	Plumbagin	[109]
*Piper longum*	Fruit and root	Alkaloid		[110]
*Pueraria tuberosa*	Tuber	β–Sitosterol, D uidzein, puerarin, isoflavone, biochanin B, puerarin, daidzein, tuberosi, stigmasterol, genistein, quercetin, irisolidone, biochanin a, isoorientin, and mangiferin	Deoxymiroestrol Genistein	[94]
*Piper nigrum*	Fruit	Aristolactams, isobutyl amide, lignin, piperine, dioxoaporphin, longamide, pluviatilol, chavicine, fargesin, asarinine		[64]
*Phyllanthus emblica*	Fruit	Chebulinic acid, gallic acid, chebulagic acid, ascorbic acid, ellagic acid, apeigenin, and quercetin	Ellagic acid and gallic acid	[111]
*Sorindeia juglandifolia* (A. Rich.)	Fruit	C-glycosylflavone, 2,3,6-trihydroxybenzoic acid, robustaflavone, 3-O-galloyl catechin, tachioside, 3]-O-D-glucopyranosyl-]-stigmasterol, 2-O-acetyl-7-O-methyl vitexin methyl gallate, 2,3,6-trihydroxymethyl benzoate mearnsitrin, 2,6-di-O-acetyl-7-O-methyl vitexin	3-O-galloyl catechin, stigmasterol, 2,3,6-trihydroxybenzoic acid	[112]
*Tiliacora triandra*	Roots	Tiliacorinine, tiliacorine, 2′-nortiliacorinine	Tiliacorine, 2′-nortiliacorinine and tiliacorinine	[113]
*Tinospora cordifolia*	Stem and roots	Berberine, tinosporoside, isocolumbin, palmarin, tinosporon, chasmanthin, tinosporic acid, and tinosporol	Not known	[95]
*Withania somnifera*	Roots	Steroidal lactones, alkaloids, withaferin A, saponins, withanone	Not known	[114]

## Data Availability

Not applicable.

## References

[1] Barberis I., Bragazzi N.L., Galluzzo L., Martini M. (2017). The history of tuberculosis: From the first historical records to the isolation of Koch’s bacillus. J. Prev. Med. Hyg..

[2] Bañuls A.L., Sanou A., Van Anh N.T., Godreuil S. (2015). *Mycobacterium tuberculosis*: Ecology and evolution of a human bacterium. J. Med. Microbiol..

[3] Montes M., Phillips C. (1959). Nonreactive tuberculosis. Am. Rev. Tuberc..

[4] Kumar P., Singh A., Sharma U., Singh D., Dobhal M.P., Singh S. (2013). Anti-mycobacterial activity of plumericin and isoplumericin against MDR *Mycobacterium tuberculosis*. Pulm. Pharmacol. Ther..

[5] Muñiz-Salazar R., Le T., Cuevas-Mota J., González-Fagoaga J.E., Zapata-Garibay R., Ruiz-Tamayo P.S., Robles-Flores J., Garfein R.S. (2022). Impact of COVID-19 on tuberculosis detection and treatment in Baja California, México. Front. Public Health.

[6] WHO (2022). Global Tuberculosis Report.

[7] Qureshi K.A., Imtiaz M., Parvez A., Rai P.K., Jaremko M., Emwas A.-H., Bholay A.D., Fatmi M.Q. (2022). In Vitro and In Silico Approaches for the Evaluation of Antimicrobial Activity, Time-Kill Kinetics, and Anti-Biofilm Potential of Thymoquinone (2-Methyl-5-propan-2-ylcyclohexa-2,5-diene-1,4-dione) against Selected Human Pathogens. Antibiotics.

[8] Rawat B., Rawat J.M., Purohit S., Singh G., Sharma P.K., Chandra A., Shabaaz Begum J.P., Venugopal D., Jaremko M., Qureshi K.A. (2022). A comprehensive review of Quercus semecarpifolia Sm.: An ecologically and commercially important Himalayan tree. Front. Ecol. Evol..

[9] Singh V.K., Mishra A., Jagannath C., Khan A., Sarwat M., Siddique H. (2022). 21—Emerging natural product based alternative therapeutics for tuberculosis. Herbal Medicines.

[10] Getachew S., Medhin G., Asres A., Abebe G., Ameni G. (2022). Traditional medicinal plants used in the treatment of tuberculosis in Ethiopia: A systematic review. Heliyon.

[11] Fatima S., Kumari A., Dwivedi V.P. (2021). Advances in adjunct therapy against tuberculosis: Deciphering the emerging role of phytochemicals. MedComm.

[12] Scarim C.B., Lira de Farias R., Vieira de Godoy Netto A., Chin C.M., Leandro Dos Santos J., Pavan F.R. (2021). Recent advances in drug discovery against *Mycobacterium tuberculosis*: Metal-based complexes. Eur. J. Med. Chem..

[13] Godreuil S., Renaud F., Van de Perre P., Carriere C., Torrea G., Banũls A.L. (2007). Genetic diversity and population structure of *Mycobacterium tuberculosis* in HIV-1-infected compared with uninfected individuals in Burkina Faso. AIDS.

[14] WHO (2019). Global Tuberculosis Report.

[15] Maus C.E., Plikaytis B.B., Shinnick T.M. (2005). Mutation of tlyA confers capreomycin resistance in *Mycobacterium tuberculosis*. Antimicrob. Agents Chemother..

[16] Muniyan R., Jayaraman G. (2017). Antimycobacterial activity of potential plant metabolites with emphasis on management of drug resistant *Mycobacterium tuberculosis* strains. Res. J. Biotechnol..

[17] Prasad K.T., Muthu V., Sehgal I.S., Dhooria S., Sharma A., Gupta N., Agarwal R. (2018). Utility of endobronchial ultrasound-guided transbronchial needle aspiration in HIV-infected patients with undiagnosed intrathoracic lymphadenopathy. Lung India.

[18] Varshney K., Anaele B., Molaei M., Frasso R., Maio V. (2021). Risk Factors for Poor Outcomes Among Patients with Extensively Drug-Resistant Tuberculosis (XDR-TB): A Scoping Review. Infect. Drug Resist..

[19] Zaman K. (2010). Tuberculosis: A global health problem. J. Health Popul. Nutr..

[20] Allué-Guardia A., García J.I., Torrelles J.B. (2021). Evolution of Drug-Resistant *Mycobacterium tuberculosis* Strains and Their Adaptation to the Human Lung Environment. Front. Microbiol..

[21] Aspatwar A., Tolvanen M.E.E., Barker H., Syrjänen L., Valanne S., Purmonen S., Waheed A., Sly W.S., Parkkila S. (2022). Carbonic anhydrases in metazoan model organisms: Molecules, mechanisms, and physiology. Physiol. Rev..

[22] Supuran C.T. (2008). Carbonic anhydrases: Novel therapeutic applications for inhibitors and activators. Nat. Rev. Drug Discov..

[23] Aspatwar A., Winum J.Y., Carta F., Supuran C.T., Hammaren M., Parikka M., Parkkila S. (2018). Carbonic Anhydrase Inhibitors as Novel Drugs against Mycobacterial beta-Carbonic Anhydrases: An Update on In Vitro and In Vivo Studies. Molecules.

[24] Aspatwar A., Kairys V., Rala S., Parikka M., Bozdag M., Carta F., Supuran C.T., Parkkila S. (2019). *Mycobacterium tuberculosis* beta-Carbonic Anhydrases: Novel Targets for Developing Antituberculosis Drugs. Int. J. Mol. Sci..

[25] Aspatwar A., Hammaren M., Parikka M., Parkkila S., Carta F., Bozdag M., Vullo D., Supuran C.T. (2020). In vitro inhibition of *Mycobacterium tuberculosis* beta-carbonic anhydrase 3 with Mono- and dithiocarbamates and evaluation of their toxicity using zebrafish developing embryos. J. Enzym. Inhib. Med. Chem..

[26] Aspatwar A., Hammaren M., Koskinen S., Luukinen B., Barker H., Carta F., Supuran C.T., Parikka M., Parkkila S. (2017). beta-CA-specific inhibitor dithiocarbamate Fc14-584B: A novel antimycobacterial agent with potential to treat drug-resistant tuberculosis. J. Enzym. Inhib. Med. Chem..

[27] Giovannuzzi S., Hewitt C.S., Nocentini A., Capasso C., Flaherty D.P., Supuran C.T. (2022). Coumarins effectively inhibit bacterial α-carbonic anhydrases. J. Enzym. Inhib. Med. Chem..

[28] Basile A., Sorbo S., Spadaro V., Bruno M., Maggio A., Faraone N., Rosselli S. (2009). Antimicrobial and antioxidant activities of coumarins from the roots of Ferulago campestris (Apiaceae). Molecules.

[29] Reen F.J., Gutiérrez-Barranquero J.A., Parages M.L., O´gara F. (2018). Coumarin: A novel player in microbial quorum sensing and biofilm formation inhibition. Appl. Microbiol. Biotechnol..

[30] Ranjita S. (2013). Nanosuspensions: A new approach for organ and cellular targeting in infectious diseases. J. Pharm. Investig..

[31] Qureshi K.A., Mohammed S.A.A., Khan O., Ali H.M., El-Readi M.Z., Mohammed H.A. (2022). Cinnamaldehyde-Based Self-Nanoemulsion (CA-SNEDDS) Accelerates Wound Healing and Exerts Antimicrobial, Antioxidant, and Anti-Inflammatory Effects in Rats’ Skin Burn Model. Molecules.

[32] Qureshi K.A., Imtiaz M., Al Nasr I., Koko W.S., Khan T.A., Jaremko M., Mahmood S., Fatmi M.Q. (2022). Antiprotozoal Activity of Thymoquinone (2-Isopropyl-5-methyl-1,4-benzoquinone) for the Treatment of Leishmania major-Induced Leishmaniasis: In Silico and In Vitro Studies. Antibiotics.

[33] Mathuria J. (2009). Nanoparticles in tuberculosis diagnosis, treatment and prevention: A hope for future. Dig. J. Nanomater. Biostructures.

[34] Nasiruddin M., Neyaz M.K., Das S. (2017). Nanotechnology-Based Approach in Tuberculosis Treatment. Tuberc. Res. Treat..

[35] Saqib S., Faryad S., Afridi M.I., Arshad B., Younas M., Naeem M., Zaman W., Ullah F., Nisar M., Ali S. (2022). Bimetallic Assembled Silver Nanoparticles Impregnated in Aspergillus fumigatus Extract Damage the Bacterial Membrane Surface and Release Cellular Contents. Coatings.

[36] El-Ridy M.S., Mostafa D.M., Shehab A., Nasr E.A., Abd El-Alim S. (2007). Biological evaluation of pyrazinamide liposomes for treatment of *Mycobacterium tuberculosis*. Int. J. Pharm..

[37] Gaspar M.M., Cruz A., Penha A.F., Reymão J., Sousa A.C., Eleutério C.V., Domingues S.A., Fraga A.G., Filho A.L., Cruz M.E. (2008). Rifabutin encapsulated in liposomes exhibits increased therapeutic activity in a model of disseminated tuberculosis. Int. J. Antimicrob. Agents.

[38] Sarciaux J.M., Acar L., Sado P.A. (1995). Using microemulsion formulations for oral drug delivery of therapeutic peptides. Int. J. Pharm..

[39] Tanwar M., Meena L.S. (2014). Nanoparticles: Scope in drug delivery. Advanced Biomaterials and Biodevices.

[40] Soppimath K.S., Aminabhavi T.M., Kulkarni A.R., Rudzinski W.E. (2001). Biodegradable polymeric nanoparticles as drug delivery devices. J. Control. Release.

[41] Satyanarayana K., Srivastava S. (2007). Poverty, health & intellectual property rights with special reference to India. Indian J. Med. Res..

[42] Kaur M., Malik B., Garg T., Rath G., Goyal A.K. (2015). Development and characterization of guar gum nanoparticles for oral immunization against tuberculosis. Drug Deliv..

[43] Taranath T.C., Patil B.N. (2016). *Limonia acidissima* L. leaf mediated synthesis of zinc oxide nanoparticles: A potent tool against *Mycobacterium tuberculosis*. Int. J. Mycobact..

[44] Murali M., Mahendra C., Nagabhushan, Rajashekar N., Sudarshana M.S., Raveesha K.A., Amruthesh K.N. (2017). Antibacterial and antioxidant properties of biosynthesized zinc oxide nanoparticles from *Ceropegia candelabrum* L.—An endemic species. Spectrochim. Acta. Part A Mol. Biomol. Spectrosc..

[45] Gupta A., Pandey S., Yadav J.S. (2021). A Review on Recent Trends in Green Synthesis of Gold Nanoparticles for Tuberculosis. Adv. Pharm. Bull..

[46] Tahir K., Nazir S., Li B., Khan A.U., Khan Z.U.H., Gong P.Y., Khan S.U., Ahmad A. (2015). Nerium oleander leaves extract mediated synthesis of gold nanoparticles and its antioxidant activity. Mater. Lett..

[47] Patra S., Mukherjee S., Barui A.K., Ganguly A., Sreedhar B., Patra C.R. (2015). Green synthesis, characterization of gold and silver nanoparticles and their potential application for cancer therapeutics. Mater. Sci. Eng. C Mater. Biol. Appl..

[48] Raju D., Vishwakarma R.K., Khan B.M., Mehta U.J., Ahmad A. (2014). Biological synthesis of cationic gold nanoparticles and binding of plasmid DNA. Mater. Lett..

[49] Bindhu M.R., Vijaya Rekha P., Umamaheswari T., Umadevi M. (2014). Antibacterial activities of Hibiscus cannabinus stem-assisted silver and gold nanoparticles. Mater. Lett..

[50] Das J., Paul Das M., Velusamy P. (2013). Sesbania grandiflora leaf extract mediated green synthesis of antibacterial silver nanoparticles against selected human pathogens. Spectrochim. Acta Part A Mol. Biomol. Spectrosc..

[51] Islam N.U., Jalil K., Shahid M., Rauf A., Muhammad N., Khan A., Shah M.R., Khan M.A. (2019). Green synthesis and biological activities of gold nanoparticles functionalized with Salix alba. Arab. J. Chem..

[52] Guo M., Li W., Yang F., Liu H. (2015). Controllable biosynthesis of gold nanoparticles from a Eucommia ulmoides bark aqueous extract. Spectrochim. Acta Part A Mol. Biomol. Spectrosc..

[53] Abdel-Raouf N., Al-Enazi N.M., Ibraheem I.B.M. (2017). Green biosynthesis of gold nanoparticles using Galaxaura elongata and characterization of their antibacterial activity. Arab. J. Chem..

[54] Philip D., Unni C. (2011). Extracellular biosynthesis of gold and silver nanoparticles using Krishna tulsi (*Ocimum sanctum*) leaf. Phys. E Low-Dimens. Syst. Nanostructures.

[55] Kalpana D., Pichiah P.B.T., Sankarganesh A., Park W.S., Lee S.M., Wahab R., Cha Y.S., Lee Y.S. (2013). Biogenesis of Gold Nanoparticles Using Plant Powders and Assessment of In Vitro Cytotoxicity in 3T3-L1 Cell Line. J. Pharm. Innov..

[56] Khalil M.M.H., Ismail E.H., El-Magdoub F. (2012). Biosynthesis of Au nanoparticles using olive leaf extract: 1st Nano Updates. Arab. J. Chem..

[57] Jha A.K., Prasad K. (2013). Rose (*Rosa* sp.) Petals Assisted Green Synthesis of Gold Nanoparticles. J. Bionanoscience.

[58] Islam N.U., Jalil K., Shahid M., Muhammad N., Rauf A. (2015). Pistacia integerrima gall extract mediated green synthesis of gold nanoparticles and their biological activities. Arab. J. Chem..

[59] Kumar K.M., Mandal B.K., Sinha M., Krishnakumar V. (2012). Terminalia chebula mediated green and rapid synthesis of gold nanoparticles. Spectrochim. Acta Part A Mol. Biomol. Spectrosc..

[60] Annamalai A., Christina V.L., Sudha D., Kalpana M., Lakshmi P.T. (2013). Green synthesis, characterization and antimicrobial activity of Au NPs using Euphorbia hirta L. leaf extract. Colloids Surfaces. B Biointerfaces.

[61] Suman T.Y., Rajasree S.R., Ramkumar R., Rajthilak C., Perumal P. (2014). The Green synthesis of gold nanoparticles using an aqueous root extract of Morinda citrifolia L.. Spectrochim. Acta Part A Mol. Biomol. Spectrosc..

[62] Sadeghi B. (2015). Zizyphus mauritiana extract-mediated green and rapid synthesis of gold nanoparticles and its antibacterial activity. J. Nanostructure Chem..

[63] Dakh K.S., Patekar R.R., Choudhary H.B., Momin A.Z., Undale V.R., Mahadik P., Desai S., Asalkar M., Shaikh H. (2022). Herbal approach for tuberculosis management: A systematic review. World J. Adv. Res. Rev..

[64] Semenya S.S., Maroyi A. (2013). Medicinal plants used for the treatment of tuberculosis by Bapedi traditional healers in three districts of the Limpopo Province, South Africa. AJTCAM.

[65] Xu Y., Liang B., Kong C., Sun Z. (2021). Traditional Medicinal Plants as a Source of Antituberculosis Drugs: A System Review. BioMed Res. Int..

[66] Bergonzi M.C., Heard C.M., Garcia-Pardo J. (2022). Bioactive Molecules from Plants: Discovery and Pharmaceutical Applications. Pharmaceutics.

[67] Goldberg G. (2003). Plants: Diet and Health: The Report of a British Nutrition Foundation Task Force.

[68] Bereda G. (2022). First Line Anti-Tuberculosis Medication: Current and Ongoing Clinical Management. J. Mol. Biol. Drug Des..

[69] Friedman N.D., McDonald A.H., Robson M.E., O'Brien D.P. (2012). Corticosteroid use for paradoxical reactions during antibiotic treatment for *Mycobacterium ulcerans*. PLoS Negl. Trop. Dis..

[70] Rahman M.A., Sobia P., Dwivedi V.P., Bhawsar A., Singh D.K., Sharma P., Moodley P., Van Kaer L., Bishai W.R., Das G. (2015). *Mycobacterium tuberculosis* TlyA Protein Negatively Regulates T Helper (Th) 1 and Th17 Differentiation and Promotes Tuberculosis Pathogenesis. J. Biol. Chem..

[71] Pakadang S.R., Wahjuni C.U., Notobroto H.B., Winarni D., Dwiyanti R., Yadi, Sabir M.M., Hatta M. (2015). Immunomodulator Potential of Miana Leaves(*Coleus scutellarioides* (L) Benth) in Prevention of Tuberculosis Infection. Am. J. Microbiol. Res..

[72] Esmaeil N., Anaraki S.B., Gharagozloo M., Moayedi B. (2017). Silymarin impacts on immune system as an immunomodulator: One key for many locks. Int. Immunopharmacol..

[73] Gupta P.K., Chakraborty P., Kumar S., Singh P.K., Rajan M.G., Sainis K.B., Kulkarni S. (2016). G1-4A, a Polysaccharide from *Tinospora cordifolia* Inhibits the Survival of *Mycobacterium tuberculosis* by Modulating Host Immune Responses in TLR4 Dependent Manner. PLoS ONE.

[74] Yan D.B., Zhang D.P., Li M., Liu W.Y., Feng F., Di B., Guo Q.L., Xie N. (2014). Synthesis and cytotoxic activity of 3, 4, 11-trihydroxyl modified derivatives of bergenin. Chin. J. Nat. Med..

[75] Dwivedi V.P., Bhattacharya D., Yadav V., Singh D.K., Kumar S., Singh M., Ojha D., Ranganathan A., Van Kaer L., Chattopadhyay D. (2017). The Phytochemical Bergenin Enhances T Helper 1 Responses and Anti-Mycobacterial Immunity by Activating the MAP Kinase Pathway in Macrophages. Front. Cell. Infect. Microbiol..

[76] Kumar S., Sharma C., Kaushik S.R., Kulshreshtha A., Chaturvedi S., Nanda R.K., Bhaskar A., Chattopadhyay D., Das G., Dwivedi V.P. (2019). The phytochemical bergenin as an adjunct immunotherapy for tuberculosis in mice. J. Biol. Chem..

[77] Eminzade S., Uraz F., Izzettin F.V. (2008). Silymarin protects liver against toxic effects of anti-tuberculosis drugs in experimental animals. Nutr. Metab..

[78] Awofeso N. (2008). Anti-tuberculosis medication side-effects constitute major factor for poor adherence to tuberculosis treatment. Bull. World Health Organ..

[79] Mariita R.M., Orodho J.A., Okemo P.O., Mbugua P.K. (2010). Antifungal, antibacterial and antimycobacterial activity of Entada abysinnica Steudel ex A. Rich (Fabaceae) methanol extract. Pharmacogn. Res..

[80] Betti J. (2013). An Ethnobotanical and Floristical Study of Medicinal Plants Among the Baka Pygmies in the Periphery of the Ipassa- Biosphere Reserve, Gabon. Eur. J. Med. Plants.

[81] Van Wyk B.E., Gericke N. (2000). People's Plants: A Guide to Useful Plants of Southern Africa.

[82] Hutchings A. (1996). Zulu Medicinal Plants: An Inventory.

[83] Green E., Samie A., Obi C.L., Bessong P.O., Ndip R.N. (2010). Inhibitory properties of selected South African medicinal plants against *Mycobacterium tuberculosis*. J. Ethnopharmacol..

[84] Breyer-Brandwijk M.G., Watt J.M. (1962). The Medicinal and Poisonous Plants of Southern and Eastern Africa being an Account of Their Medicinal and Other Uses, Chemical Composition, Pharmacological Effects and Toxicology in Man and Animal.

[85] Maroyi A. (2011). An ethnobotanical survey of medicinal plants used by the people in Nhema communal area, Zimbabwe. J. Ethnopharmacol..

[86] Abubakar E.-M.M. (2010). Antibacterial potential of crude leaf extracts of Eucalyptus camaldulensis against some pathogenic bacteria. Afr. J. Plant Sci..

[87] Moeng T.E. (2010). An Investigation into the Trade of Medicinal Plants by Muthi Shops and Street Vendors in the Limpopo Province, South Africa. Ph.D. Thesis.

[88] Grierson D.S., Afolayan A.J. (1999). An ethnobotanical study of plants used for the treatment of wounds in the Eastern Cape, South Africa. J. Ethnopharmacol..

[89] Eldeen I.M.S., Van Staden J. (2007). Antimycobacterial activity of some trees used in South African traditional medicine. S. Afr. J. Bot..

[90] Bryant A.Y. (1966). Zulu Medicine and Medicine Men.

[91] Shang A., Cao S.Y., Xu X.Y., Gan R.Y., Tang G.Y., Corke H., Mavumengwana V., Li H.B. (2019). Bioactive Compounds and Biological Functions of Garlic (*Allium sativum* L.). Foods.

[92] Malebo H.M., Wenzler T., Cal M., Swaleh S.M., Omolo M.O., Hassanali A., Séquin U., Häussinger D., Dalsgaard P., Hamburger M. (2013). Anti-protozoal activity of aporphine and protoberberine alkaloids from Annickia kummeriae (Engl. & Diels) Setten & Maas (Annonaceae). BMC Complement. Altern. Med..

[93] León-Díaz R., Meckes-Fischer M., Valdovinos-Martínez L., Campos M.G., Hernández-Pando R., Jiménez-Arellanes M.A. (2013). Antitubercular activity and the subacute toxicity of (-)-Licarin A in BALB/c mice: A neolignan isolated from Aristolochia taliscana. Arch. Med. Res..

[94] Samal J. (2016). Ayurvedic management of pulmonary tuberculosis: A systematic review. J. Intercult. Ethnopharmacol..

[95] Gupta R., Thakur B., Singh P., Singh H.B., Sharma V.D., Katoch V.M., Chauhan S.V. (2010). Anti-tuberculosis activity of selected medicinal plants against multi-drug resistant *Mycobacterium tuberculosis* isolates. Indian J. Med. Res..

[96] Balážová Ľ., Kurhajec S., Kello M., Bedlovičová Z., Zigová M., Petrovová E., Beňová K., Mojžiš J., Eftimová J. (2022). Antiproliferative Effect of Phellodendron amurense Rupr. Based on Angiogenesis. Life.

[97] Changtam C., Hongmanee P., Suksamrarn A. (2010). Isoxazole analogs of curcuminoids with highly potent multidrug-resistant antimycobacterial activity. Eur. J. Med. Chem..

[98] Torres-Romero D., Jiménez I.A., Rojas R., Gilman R.H., López M., Bazzocchi I.L. (2011). Dihydro-β-agarofuran sesquiterpenes isolated from *Celastrus vulcanicola* as potential anti-*Mycobacterium tuberculosis* multidrug-resistant agents. Bioorganic Med. Chem..

[99] Uc-Cachón A.H., Borges-Argáez R., Said-Fernández S., Vargas-Villarreal J., González-Salazar F., Méndez-González M., Cáceres-Farfán M., Molina-Salinas G.M. (2014). Naphthoquinones isolated from *Diospyros anisandra* exhibit potent activity against pan-resistant first-line drugs *Mycobacterium tuberculosis* strains. Pulm. Pharmacol. Ther..

[100] Esquivel-Ferriño P.C., Favela-Hernández J.M., Garza-González E., Waksman N., Ríos M.Y., del Rayo Camacho-Corona M. (2012). Antimycobacterial activity of constituents from *Foeniculum vulgare* var. dulce grown in Mexico. Molecules.

[101] Ogodo A.C., Ebuara F.U., Ogodo C.F. (2021). Bacteriocidal Effects of Phytochemicals on Mycobacterium ulcerans, the Causative Agents of Buruli Ulcer. Negl. Trop. Dis. Phytochem. Drug Discov..

[102] Molina-Salinas G.M., Peña-Rodríguez L.M., Mata-Cárdenas B.D., Escalante-Erosa F., González-Hernández S., Torres de la Cruz V.M., Martínez-Rodríguez H.G., Said-Fernández S. (2011). *Flourensia cernua*: Hexane Extracts a Very Active Mycobactericidal Fraction from an Inactive Leaf Decoction against Pansensitive and Panresistant *Mycobacterium tuberculosis*. Evid. Based Complement. Altern. Med. Ecam.

[103] Kapche G.D.W.F., Laatsch H., Fotso S., Kouam S.F., Wafo P., Ngadjui B.T., Abegaz B.M. (2007). Lanneanol: A new cytotoxic dihydroalkylcyclohexenol and phenolic compounds from *Lannea nigritana* (Sc. Ell.) Keay. Biochem. Syst. Ecol..

[104] Shingate P., Dongre P.P., Kannur D.M. (2013). New Method Development for Extraction and Isolation of Piperine from Black Pepper. Int. J. Pharm. Sci. Res..

[105] Tandon N., Yadav S.S. (2017). Contributions of Indian Council of Medical Research (ICMR) in the area of Medicinal plants/Traditional medicine. J. Ethnopharmacol..

[106] Jayapal V., Vidya Raj C.K., Muthaiah M., Chadha V.K., Brammacharry U., Selvaraj S., Easow J.M. (2021). In-vitro anti-*Mycobacterium tuberculosis* effect of essential oil of *Ocimum sanctum* L. (Tulsi/Basil) leaves. Indian J. Tuberc..

[107] Udegbunam S.O., Udegbunam R.I., Muogbo C.C., Anyanwu M.U., Nwaehujor C.O. (2014). Wound healing and antibacterial properties of methanolic extract of *Pupalia lappacea* Juss in rats. BMC Complement. Altern. Med..

[108] Rijo P., Simões M.F., Francisco A.P., Rojas R., Gilman R.H., Vaisberg A.J., Rodríguez B., Moiteiro C. (2010). Antimycobacterial metabolites from *Plectranthus*: Royleanone derivatives against *Mycobacterium tuberculosis* strains. Chem. Biodivers..

[109] Sarkar A., Ghosh S., Shaw R., Patra M.M., Calcuttawala F., Mukherjee N., Das Gupta S.K. (2020). *Mycobacterium tuberculosis* thymidylate synthase (ThyX) is a target for plumbagin, a natural product with antimycobacterial activity. PLoS ONE.

[110] Xiao Y., Shi M., Qiu Q., Huang M., Zeng S., Zou Y., Zhan Z., Liang L., Yang X., Xu H. (2016). Piperlongumine Suppresses Dendritic Cell Maturation by Reducing Production of Reactive Oxygen Species and Has Therapeutic Potential for Rheumatoid Arthritis. J. Immunol..

[111] Starke J.R. (1988). Modern approach to the diagnosis and treatment of tuberculosis in children. Pediatr. Clin. N. Am..

[112] Ndongo J.T., Mbing J.N., Bikobo D.N., Atchadé A.T., Shaaban M., Pegnyemb D.E., Laatsch H. (2013). A new C-glucosylflavone from Sorindeia juglandifolia. Z. Fur Naturforschung. C J. Biosci..

[113] Sureram S., Senadeera S.P., Hongmanee P., Mahidol C., Ruchirawat S., Kittakoop P. (2012). Antimycobacterial activity of bisbenzylisoquinoline alkaloids from *Tiliacora triandra* against multidrug-resistant isolates of *Mycobacterium tuberculosis*. Bioorganic Med. Chem. Lett..

[114] Mueller-Wieland K. (1961). Tuberculosis and nutrition. Dtsch. Med. J..

[115] Xu Z.-Q., Barrow W.W., Suling W.J., Westbrook L., Barrow E., Lin Y.-M., Flavin M.T. (2004). Anti-HIV natural product (+)-calanolide A is active against both drug-susceptible and drug-resistant strains of *Mycobacterium tuberculosis*. Bioorganic Med. Chem..

